# First isolation, identification and genetic characterization of *Brucella abortus* biovar 3 from dairy cattle in Bangladesh

**DOI:** 10.1002/vms3.193

**Published:** 2019-08-26

**Authors:** Md. Sadequl Islam, Giuliano Garofolo, Lorena Sacchini, Amanda C. Dainty, Mst. Minara Khatun, Sukumar Saha, Md. Ariful Islam

**Affiliations:** ^1^ Department of Microbiology and Hygiene Bangladesh Agricultural University Mymensingh Bangladesh; ^2^ Hajee Mohammad Danesh Science and Technology University Dinajpur Bangladesh; ^3^ Istituto Zooprofilattico Sperimentale dell'Abruzzo e del Molise "G. Caporale" Teramo Italy; ^4^ Animal and Plant Health Agency Weybridge UK

**Keywords:** Bangladesh, biotyping, *Brucella abortus* biovar 3, Dairy cattle, genetic characterization, MLVA‐16

## Abstract

**Background:**

Brucellosis is a zoonotic disease caused by bacteria *Brucella* spp. belonging to the genus *Brucella*. It is endemic in domesticated animals in Bangladesh. Isolation, identification and genetic characterization of *Brucella* spp. in dairy cattle are essential to undertake appropriate control and preventive measures. The study was conducted to isolate and characterize the *Brucella* spp. circulating in dairy cattle.

**Methods:**

Uterine discharge (*n* = 45), milk (*n* = 115), vaginal swab (*n* = 71), placenta (*n* = 7) and aborted fetus (*n* = 2) were collected. Brucella selective agar plates were inoculated with samples and incubated at 37 ^◦^C for 14 days under 5% CO_2_ for isolation of *Brucella* spp. *Brucella* suspected colonies were recovered from samples were confirmed by genus and species specific PCR assays. Genetic characterization was performed by Multi Locus Variable number tandem‐repeat Analysis‐16 (MLVA‐16).

**Results:**

The isolates of *Brucella* recovered from samples were confirmed as *B. abortus* by AMOS‐ERY PCR assay. The classical biotyping method confirmed all 10 *B. abortus* isolates belonged to the biovar 3. The MLVA‐16 assay indicated all *B. abortus* isolates identical and the same genotype 40, based on panel 1 MLVA‐8.

**Conclusion:**

Dendrogram analysis revealed all *B. abortus* isolates of the study were identical to three isolates from Brazil, one isolate of France and closely related to Chinese isolates. This is the first report of isolation and genetic characterization of *B. abortus* from the dairy cattle in Bangladesh.

## Abstract 2

This study isolates and identifies 10 *Brucella abortus* biovar 3 from the dairy cattle experiencing abortion. Multi Locus Variable number tandem‐repeat Analysis‐16 (MLVA‐16) revealed all *B. abortus* isolates of the study were identical to three isolates from Brazil, one isolate of France and closely related to Chinese isolates. This is the first report of isolation and genetic characterization of *B. abortus* biovar 3 from the dairy cattle in Bangladesh.

## Graphical abstract

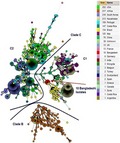

## 1 INTRODUCTION

Brucellosis is a worldwide zoonotic infection of economic and public health importance caused by bacteria from genus *Brucella* (Mathew et al., 2015). These are non‐motile, facultative anaerobic, intracellular, Gram‐negative coccobacilli and different species show strong host specificity (Pappas, Akritidis, Bosilkovski, & Tsianos, 2005). There are five species of *Brucella* known to cause diseases in domesticated animals: *B. abortus* (cattle), *B. melitensis* (goats), *B. ovis* (sheep), *B. suis* (pigs) and *B. canis* (dogs). *B. abortus* has been subdivided into biovars 1, 2, 3, 4, 5, 6, 7 and 9. The biovar 3 consists of two genetically disparate sub‐groups 3a and 3b (Ocampo‐Sosa, Aguero‐Balbin, & Garcia‐Lobo, 2005). Four distinct clades of *B. abortus* have been proposed: clades A, B and C (C1 and C2) to show the intraspecies relationships among its biovars (Whatmore et al., 2016).

Brucellosis causes abortion, infertility, still birth and reduced milk production in animals. Animals get infected either through consumption of contaminated feed and water or contact with an infected animal. Routine bacteriological method and a classical biotyping scheme are used for characterization of *Brucella* both at species and subspecies levels (Alton, Jones, & Pietz, 1975; Whatmore et al., 2016). Polymerase chain reaction (PCR) assays are currently used for identification of *Brucella* at genus, species and biovar levels (Bricker & Halling, 1994; Ocampo‐Sosa et al., 2005; Romero, Gamazo, & Pardo, 1995). Multi Locus Variable number tandem‐repeat Analysis (MLVA) assay is used for genetic characterization of *Brucella* isolates (Le Fletch et al., 2006; Whatmore et al., 2006).

Brucellosis has been reported in humans and animals in Bangladesh (Islam, Khatun, Werre, Sriranganathan, & Boyle, 2013; Rahman et al., 2012). It is known to cause huge economic losses in livestock sector (Islam et al., 2013; Rahman, Choudhury, Rahman, & Haque, 1983). Livestock farmers, butchers, milkers and veterinarians are high risk group individuals to contract brucellosis (Rahman et al., 2012). A seroprevalence report of brucellosis in Bangladesh listed a 3.7% prevalence of brucellosis in cattle, 4% in buffalo, 3.6% in goats and 7.3% in sheep (Islam et al., 2013).

Isolation of *Brucella* at the genus level has been reported in milk sample of cattle (Islam et al., 2018). *Brucella* genus‐specific DNA has been identified in the sera of humans by real time PCR assay (Rahman et al., 2012). The *B. aborus* species‐specific DNA has been detected in the sera of cattle by real time PCR (Rahman et al., 2014). Isolation of *Brucella* from the infected host is considered as the gold standard for diagnosis of brucellosis (Rahman et al., 2012). However, identification of *Brucella* at species level and its biovar typing and genetic characterization of circulating *Brucella* spp. has not been reported in Bangladesh. The objectives of the present research work are: i) isolation of *Brucella* spp. from dairy cattle experiencing abortion, ii) identification of *Brucella* at species and biovar levels and iii) genetic characterization of circulating *Brucella* spp. by MLVA‐16 assay.

## 2 MATERIALS AND METHODS

### 2.1 Study areas and samples

**Figure 1:**
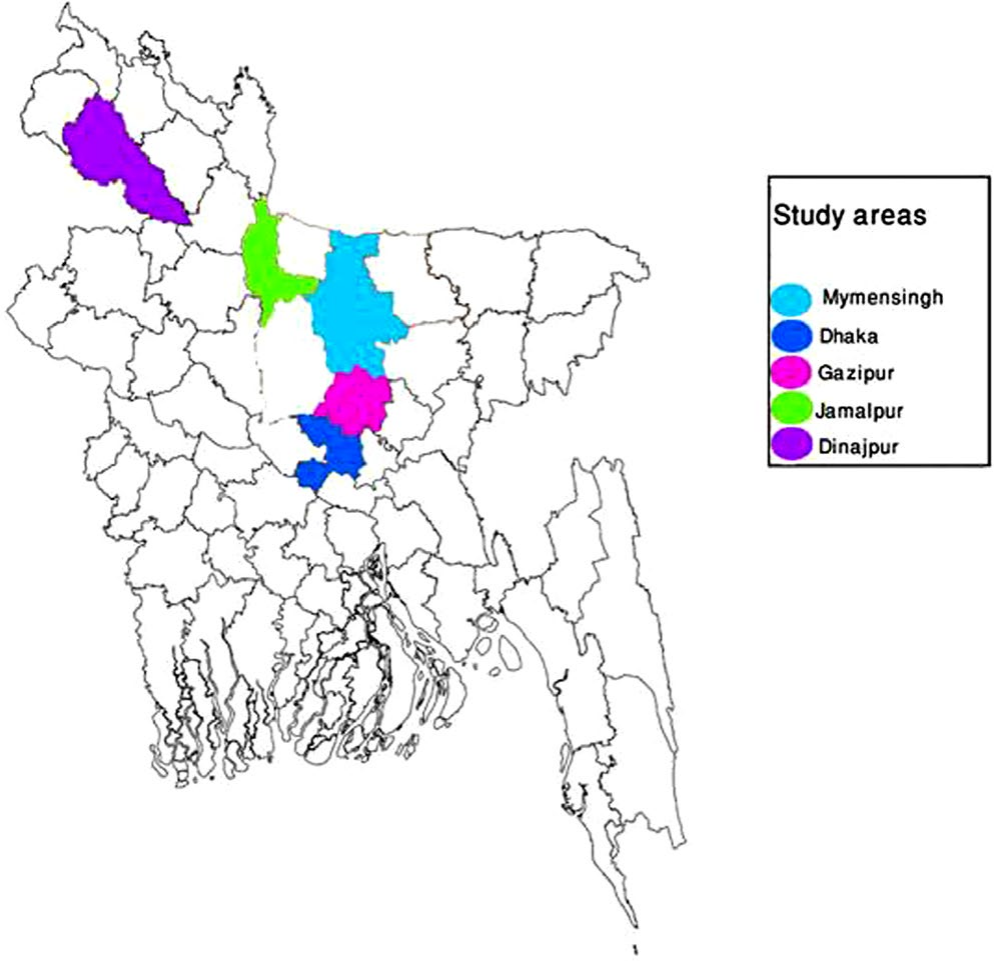
Map of Bangladesh indicating study areas by colour highlights

The study was conducted in 1,285 dairy cattle on 22 farms located in the following geographical areas of Bangladesh: Mymensingh Sadar (24.7500ºN 90.4167ºE), Dhaka Savar (23.8583ºN 90.2667ºE), Gazipur Sadar (24.0000ºN 90.4250ºE), Jamalpur Sadar (24.9167ºN 89.9583ºE) and Dinajpur Sadar (25.5856ºN 88.6531ºE) (Figure 1). Each of the dairy farm consisted of 20–100 cattle which were indigenous breed and crossbreed of Friesian, Sahiwal and Red Chittagong. The cattle of the study farms were not vaccinated against brucellosis. A total of 240 samples consisted of uterine discharges (*n* = 45), milk (*n* = 115), vaginal swabs (*n* = 71), placenta (*n* = 7) and aborted fetuses (*n* = 2) were collected from dairy cattle with the clinical sign of abortion (*n* = 55) or without the history of abortion (*n* = 185) during the period from August 2016 to December 2017. Samples were collected after 1 to 3 days of abortion. The abortion was occurred at third trimester of gestation from first to third pregnancy. Uterine discharge (10 ml) was collected by inserting a disposable artificial insemination pipette into the uterus. Midstream milk sample (20 ml) was collected from each quarter of the udder into a sterile 50 ml falcon tube. Vaginal swab was collected using a sterile applicator stick (HiMedia,, Mumbai, India). Placental cotyledons were collected from aborted cattle aseptically using sterile forceps and scissors and kept in a sterile plastic container. The aspirate of stomach content (50ml) was collected from the aborted fetus. The samples were transported to the Department of Microbiology and Hygiene, Bangladesh Agricultural University, using an ice box and kept at 4°C and cultured within 3 days.

### 2.2 Isolation and biotyping of bacteria

Uterine discharge, vaginal swab and aspirate of fetal stomach content were streaked duplicate onto the Brucella selective agar supplemented with antibiotics (polymyxin B sulphate, bacitracin, nystatin, cycloheximide, nalidixic acid, vancomycin) (HiMedia, Mumbai, India) that inhibit growth of bacteria other than *Brucella* (Alton, Jones, Angus, & Veger, 1988). Milk was centrifuged at 3500rpm for 15 min. The cream and sediment were inoculated onto the Brucella selective agar (HiMedia, Mumbai, India) using a sterile cotton swab. Placental cotyledons were cut into small pieces and placed in a sterile plastic bag with equal volume of phosphate‐buffered saline. The cotyledons were macerated by a stomacher for 5 min and tissue homogenate was inoculated onto the Brucella selective agar (HiMedia, Mumbai, India) using a sterile cotton swab. Inoculated plates were placed in an incubator supplied with 5% CO_2_ at 37°C. The plates were observed daily up to 14 days for *Brucella* like colonies (smooth, small, translucent, glistening, dew drop like round and convex colony). Identification of bacteria in pure culture was performed by colony morphology, Gram's staining reaction, catalase, oxidase, H_2_S and urease tests (Alton et al., 1975).

*Brucella* spp. were subjected to classical biotyping described by Alton et al. (1988). A panel of biotyping tests such as CO_2_ requirement for growth, H_2_S production and growth in presence of thionine and basic fuchsin were performed.

### 2.3 Molecular identification and genotyping of *B. abortus*

The genomic DNA was extracted from suspect *Brucella* colonies by a genomic DNA extraction kit using manufacturer's protocol (GeneJet Genomic DNA Purificaion Kit, Thermo Fisher Scientific, Vilnius, Lithuania).

To confirm *Brucella* spp. at molecular level a genus specific PCR assay targeting 905 bp fragment of the 16S *rRNA* gene was performed (Romero et al., 1995). Identification of *B. abortus* biovar 1, 2 and 4 was performed by AMOS PCR assay with oligonucleotide primers and PCR conditions described by Bricker and Halling (1994). Enhanced AMOS‐ ERY PCR assay was performed for the detection of *B. abortus* biovar 3b, 5, 6 and 9 (Ocampo‐Sosa et al., 2005) with modification of the annealing temperature. The MLVA genotyping was done at the OIE Reference laboratory for Brucellosis: Istituto Zooprofilattico Sperimentale dell'Abruzzo e del Molise "G. Caporale", Teramo, Italy. Samples were genotyped using the MLVA‐16 panel (Le Fleche et al., 2006), with modifications (Al Dahouk et al., 2007). Loci considered were Bruce 06, Bruce 08, Bruce 11, Bruce 12, Bruce 42, Bruce 43, Bruce 45, Bruce 55, Bruce 18, Bruce 19, Bruce 21, Bruce 04, Bruce 07, Bruce 09, Bruce 16 and Bruce 30.

Amplification of MLVA‐16 loci was performed using multiplex PCRs as described previously (Garofolo, Ancora, & Giannatale, 2013). PCR amplifications were performed in a total volume of 10 μl containing 1.50 ng DNA, 1 × Type‐it microsatellite PCR Master Mix (QiagenSrl, Milan, Italy), and proper concentration of each fluorescent primer pairs (Garofolo et al., 2013). Thermal cycling was conducted on a GeneAmp 9700 thermal cycler (Applied Biosystems) following thermal reaction profiles: initial heating at 95°C for 5 min, 30 cycles denaturation at 95°C for 30 s, annealing at 60°C for 90 s and extension at 72°C for 30 s. A final extension step at 60°C for 45 min and 20°C for 120 min was run to reduce artefacts such as stutter and non‐templated 3' A nucleotide additions. Fragments were then separated through capillary electrophoresis on an ABI 3500 instrument with POP 7 polymer. Data analysis was done using Genemapper 4.1 (Applied Biosystems) to assign for each VNTRs the actual allele. Clustering analyses were conducted with BioNumerics 6.6 (Applied‐Maths) accessing additional data from the international MLVA Database (http://mlva.u-psud.fr/mlvav4/genotyping/) and treating as a character dataset with the categorical distance coefficient and UPGMA (Unweighted Pair‐Group Method Arithmetic Average) and MST (Minimum Spanning Tree) methods. The MST for single clade retrieved the clonal complexes with the most stringent (conservative) definition, where all members assigned to the same group differ only at one locus format least one other member of the group.

## 3 RESULTS

### 3.1 Isolation and biotyping characteristics

Ten *Brucella* spp. were isolated from uterine discharge (*n* = 7, sample ID no. 21/S‐4023, 46/S‐5083, 49/G‐7, 66/G‐12, 106/G‐5213, 107/S‐55 and 109/S‐1978), milk (*n* = 2, sample ID no. 72/ G‐22 and 84/S‐756) and vaginal swab (*n* = 1, sample ID no. 61/S‐1000) of 10 dairy cows that suffered an abortion at the third trimester of pregnancy. They grew in a 5% CO_2_ atmosphere after 3–14 days incubation at 37°C. Bacterial colonies were small, convex and regular with smooth surface, honey coloured, shiny and translucent. The organisms appeared to be Gram negative, small coccobacilli arranged singly or in pairs. The isolates were catalase, oxidase, H_2_S and urease positive. The isolates grew in the presence of thionin and basic fuchsin dyes suggesting that all isolates belonged to the biovar 3 (Table 1).

**Table 1:** Biotyping results of *Brucella abortus* isolated from dairy cattle

No. of *B. abortus* isolates tested	Growth Characteristics									Interpretation
	Urease activity	CO_2_ requirement	H_2_S production	Serum requirement	Thionin blue*			Basic fuchsin**		
					a	b	c	b	c	
10	**+**	**+**	**+**	**‐**	**+**	**+**	**+**	**+**	**+**	*B. abortus* biovar 3

Note

+ = Positive, ‐ = Negative, *Concentration of thionin blue (a = 1:25,000, b = 1:50,000, c = 1:10,0000), **Concentration of basic fuchsin (b = 1:50,000, c = 1:10,0000)

### 3.2 Molecular identification by PCR

*Brucella* genus specific PCR targeting 16S *rRNA* gene amplified 905 bp size of PCR amplicons. It confirmed the identity of the 10 isolates as *Brucella*. The enhanced AMOS‐ ERY PCR reconfirmed all isolates as *B. abortus* with the production of 1,700 bp size PCR amplicons.

### 3.3 MLVA genotyping

**Figure 2:**
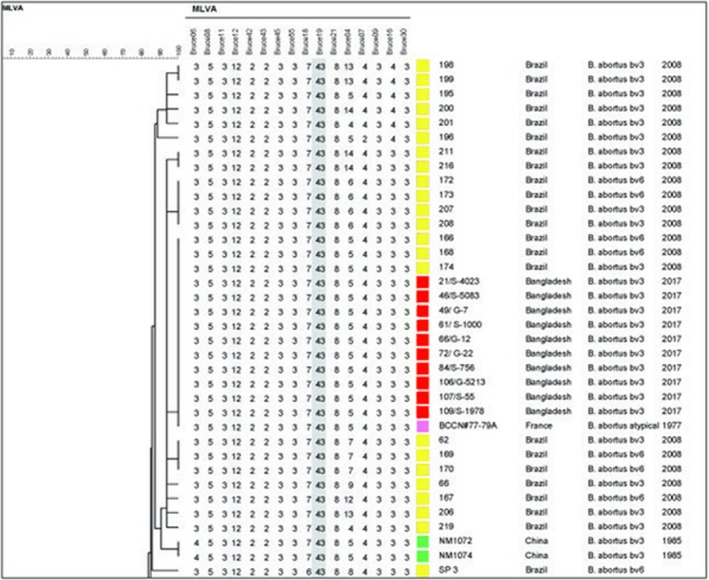
An extract from an unweighted dendrogram constructed from the profiles of 1633 *Brucella* isolates submitted to the international MLVA database (MLVA‐NET) using UPGMA analysis, categorical coefficient plus the 10 isolates from Bangladesh. The MLVA‐16 profiles of the 10 isolates from Bangladesh are shown to cluster with isolates from Brazil. The columns following the data represent sample ID, country of origin, species/biovar and year of isolation. The coloured boxes denote country of origin

**Figure 3:**
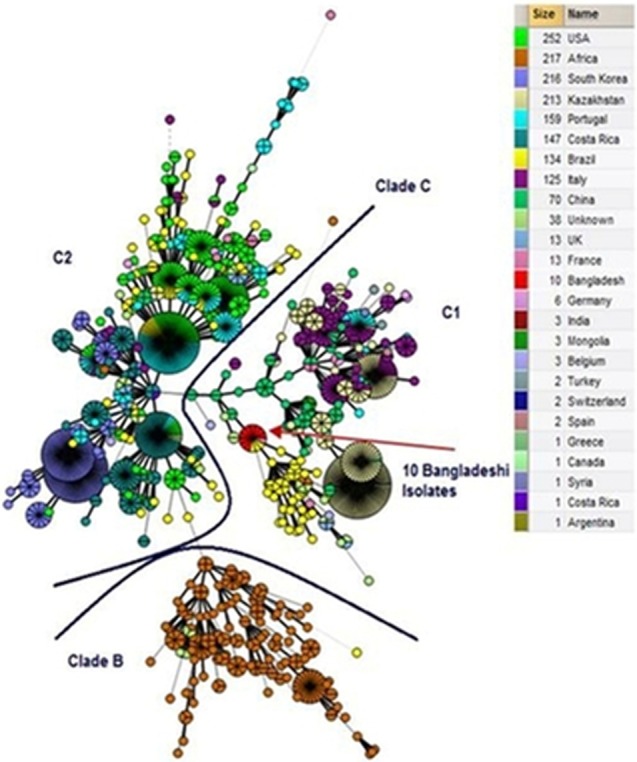
A minimum spanning tree (MST) was constructed in BioNumerics 6.6 using the categorical coefficient and default settings to examine the clustering of the *B. aborus* isolates from Bangladesh on a global scale. The MST was split by country/continent and colour coded accordingly. The data were compared against the MLST clades, where the 1633 *B. aborus* isolates examined corresponded to three clades/subclades described previously, namely C1, C2 and B. No isolates of the rare clade A, associated with Africa, are included in the international MLVA database. The 10 *B. aborus* isolates from Bangladesh fell in clade C1 indicated by a red arrow. Name denotes country of origin of the isolates and size denotes number of isolates evaluated. Each circle represents a unique genotype. The diameter of each circle corresponds to the number of isolates with the same genotype and its size is proportional to the number of strains

The dendrogram analysis revealed the 10 isolates from Bangladesh shared the same genotype 40, based on Panel 1 MLVA 8 and were identical to three isolates from Brazil and one isolate from France (Figure 2). A further seven isolates from Brazil clustered closely, differing only by one highly polymorphic minisatellite from Panel 2B, (Bruce 04). Two isolates from China were also found within the same branch, differing by one locus from Panel 1, (Bruce 06), where known, all closely related isolates were reported as members of biovar 3 or biovar 6. The 10 isolates from Bangladesh fell within Clade C1 (Figure 3). All MLVA profiles have been submitted to the MLVA bank (http://microbesgenotyping.i2bc.paris-saclay.fr/).

## 4 DISCUSSION

Brucellosis causes abortion in the third trimester of bovine pregnancy (Megid, Mathias, & Robles, 2010). The *Brucella* are known to be shed in the aborted materials of cattle such as: uterine discharge, vaginal swab, placenta and fetus. In the present study, *B. abortus* was isolated from the aborted materials of dairy cattle at the third trimester of gestation. *B. aborus* was not isolated from the stomach content of aborted fetus. This may be either due to absence of bacteria in the stomach content or low number of samples tested. Similar results were also reported by Geresu, Ameni, Wubete, Arenas‐Gamboa, and Kassa (2016). The current research work also isolated *B. abortus* from the milk of dairy cattle that had aborted. *Brucella* is known to shed from the milk of infected cattle (Capparelli et al., 2009). In brucellosis endemic areas, transmission of *Brucella* to humans can occur through consumption of unpasteurized milk (Deshmukh et al., 2015). In Bangladesh milk ring tests are not routinely practiced for screening *B. abortus* specific antibodies in milk (Islam et al., 2018). The presence of *B. abortus* in cattle milk constitutes a public health hazard as people in Bangladesh mostly purchase unpasteurized milk.

In the study, AMOS PCR assay failed to amplify a 498 bp PCR amplicon (data not shown) indicating none of the *Brucella* isolates belonged to the *B. abortus* biovar 1, 2 and 4 (Bricker & Halling, 1994). The AMOS‐ ERY assay identifies *B. abortus* biovar 3b, 5, 6 and 9 (Ocampososa et al., 2005) suggesting that the *B. abortus* isolate*s* from cattle might be any one of these four biovars.

In the present study, all *B. abortus* isolates of cattle belonged to the biovar 3*,* indicating this biovar is being transmitted in the dairy cattle in the study areas. In this study, *B. abortus* was isolated from dairy farms located in two neighbouring districts of Bangladesh; Dhaka (Savar) and Gazipur. Therefore, it is very difficult to draw a conclusion that the *B. abortus* biovar 3 is predominately circulating in dairy population in Bangladesh as this study screened only a small number of samples obtained from aborted cows in a limited geographical area of Bangladesh. All isolates were designated MLVA panel‐1 genotype 40 and had full MLVA‐16 profiles identical to three isolates from Brazil (Minharro et al., 2013) and one isolate from France (Vergnaud et al., 2018). While Bangladesh and Brazil are clearly geographically well separated, it has previously been reported that most of the cattle imported into Brazil are from Europe or India (Minharro et al., 2013). This provides a plausible explanation for the sharing of MLVA‐16 profiles between Brazil and Bangladesh, which borders India. The MLVA data, compared against the multilocus sequence typing (MLST) clades proposed by Whatmore et al. (2016), showed the isolates fell within Clade C1. In contrast with clade B, associated almost exclusively with isolates from Africa, this clade has a global distribution. Within clade C1, the most common biovar association is with biovar 3, which is consistent with the results obtained in this study. Biovar 3 is known to consist of at least two major genetically disparate groups (Ocampo‐Sosa et al., 2005; Whatmore et al., 2016) and the isolates described here correspond to sub‐group 3b of *B. abortus* biovar 3, more commonly of European origin than sub‐group 3a associated with African origins.

Similarly, while all isolates shared an identical MLVA profile, much more sampling is required to understand the local diversity of *Brucella*, and whether sufficient diversity exists such that MLVA may be a useful tool to inform understanding of epidemiological linkages locally. Isolates were identical to some previously reported from Brazil and closely related to others from China (Jiang et al., 2013) whether these represent true epidemiological linkages or simply homoplasy remains unclear. Further analysis with approaches such as whole genome sequencing would help to more categorically establish the relationship of these isolates with the global population. Characterization of the species and biovars of *Brucella* from field outbreak and to trace back the source of new strain in a particular geographic area is important to undertake effective control measures against brucellosis (De Massis et al., 2019). The data of species and biovar identification and genetic characterization of *Brucella* field isolates of the present work may be useful to formulate policy and strategies for the control of bovine brucellosis in Bangladesh.

## CONFLICT OF INTEREST

The authors declare no competing interest.

## ETHICAL STATEMENT

The protocol for field studies and collection of animal sample was approved by Animal Welfare and Ethical Committee, Bangladesh Agricultural University, Mymensingh‐2202. Farmers were informed and their verbal consent was taken previously for the collection of samples from their animals.
